# Digital technologies for non-invasive stress detection, monitoring, and mitigation in children and adolescents: a scoping review

**DOI:** 10.3389/fdgth.2026.1867488

**Published:** 2026-07-13

**Authors:** Arshad Nasser, Malak Baslyman, Sami Elferik

**Affiliations:** King Fahd University of Petroleum and Minerals, Dhahran, Saudi Arabia

**Keywords:** adolescent, child, mHealth, non-invasive monitoring, pediatric stress, telehealth, wearable technology

## Abstract

**Background:**

Stress in childhood and adolescence can disrupt emotional, cognitive, and physiological development and contribute to long-term health consequences. Conventional assessments, such as cortisol sampling and retrospective questionnaires, are often invasive, burdensome, and poorly suited for repeated pediatric use. Advances in mobile health, wearable devices, and other non-invasive sensing approaches offer new opportunities for real-time stress detection, monitoring, and mitigation in children and adolescents.

**Objective:**

This scoping review synthesizes evidence on non-invasive technologies for pediatric stress detection, monitoring, and mitigation, with attention to mobile, wearable, and ubiquitous health applications. It maps reported technologies, protocols, study contexts, and intervention approaches, and identifies gaps for future research and practice.

**Methods:**

Following PRISMA-ScR guidelines, four bibliographic databases–PubMed, Scopus, IEEE Xplore, and ACM Digital Library–were searched, with Google Scholar screened as a supplementary source. Searches covered peer-reviewed studies published from January 2013 to April 2026. Eligible studies involved participants aged 0–18 years, evaluated non-invasive technological methods for stress detection, monitoring, or mitigation, and reported empirical outcomes. Data were charted across technology type, stress induction and validation, study context, age group, evaluation method, datasets, and intervention strategies.

**Results:**

Thirty-four studies were included. Wearable and mobile sensing approaches dominated the literature, while fewer studies examined non-contact, ambient, or hybrid systems. Stress induction and assessment methods were heterogeneous, often relying on task-based, situational, or context-specific stressors rather than standardized pediatric protocols. Studies were conducted in school, laboratory, home, therapy, and emerging clinical contexts, although longitudinal real-world deployments remained limited. Children under 13 years were more frequently represented than adolescents, while younger children and developmentally diverse populations remained unevenly studied. A smaller subset examined mitigation strategies, including biofeedback, serious games, smart objects, and caregiver- or parent-mediated interventions. Ethical, privacy, and implementation issues were acknowledged but not systematically addressed.

**Conclusions:**

Non-invasive technologies show strong potential for pediatric stress detection, monitoring, and mitigation, particularly through wearable, mobile, and interactive systems. However, the field remains methodologically fragmented. Future work should develop age-appropriate stress protocols, improve dataset diversity and transparency, conduct ecologically valid longitudinal evaluations, and strengthen ethical, privacy, and equity considerations for translation into schools, homes, telehealth, and clinical care.

## 1 Introduction

Stress during childhood and adolescence can shape emotional, cognitive, behavioral, and physiological development, and persistent or poorly managed stress has been linked to adverse long-term health outcomes [1–3]. Early identification and support are therefore important during these developmental stages.

Conventional approaches to stress assessment have often relied on invasive biomarkers, laboratory procedures, or retrospective psychometric instruments, such as cortisol sampling and self-report questionnaires administered after the stressful event [4]. Although such methods can be informative, they present practical, ethical, and methodological challenges in pediatric populations, where repeated invasive assessment, long testing procedures, or recall-dependent methods may be burdensome and developmentally inappropriate.

Recent technological advances have increased interest in non-invasive approaches for pediatric stress detection, monitoring, and mitigation. These include wearable sensing systems, mobile and mHealth platforms, interactive biofeedback tools, smart objects, and a smaller number of contactless or ambient sensing approaches. Such systems commonly use physiological signals such as heart rate, heart rate variability, electrodermal activity, skin temperature, respiration, and movement, and in some cases also incorporate behavioral indicators such as speech, facial activity, gaze, or contextual interaction patterns. Their use in schools, homes, laboratories, therapy settings, and emerging clinical environments raises important questions about scalability, age-appropriateness, and ecological validity that this review sets out to address.

Despite this growing interest, the literature remains fragmented. Pediatric studies vary considerably in sensing modality, stress induction protocol, validation strategy, study context, target age group, and evaluation framework. Many systems are tested in small samples or narrowly defined settings, making it difficult to compare findings across studies or assess translational readiness. In addition, children and adolescents differ from adults in their developmental profiles, stress responses, dependence on caregivers, and ethical protection needs, limiting the direct transferability of adult-oriented stress technologies to pediatric populations.

Given these challenges, there is a clear need to map the current state of non-invasive pediatric stress technologies in a structured way. Beyond identifying which technologies are being used, it is also necessary to understand what signals they assess, how stress is induced or validated, where and with whom these systems are deployed, how performance is evaluated, what datasets are available, and how mitigation or intervention components are incorporated.

This scoping review addresses that need by synthesizing literature focused explicitly on non-invasive technologies for stress detection, monitoring, and mitigation in children and adolescents. Although the included systems vary in form, most are implemented through wearable, mobile, and interactive platforms, with fewer examples of ambient or contactless sensing. Specifically, this review aims to: (1) identify the technologies and physiological or behavioral parameters assessed; (2) examine methods used to induce, measure, or validate stress in pediatric studies; (3) explore the settings and age groups in which these technologies have been applied; (4) review the detection approaches, evaluation frameworks, and reported performance metrics used across studies; (5) summarize available pediatric datasets and their characteristics; (6) examine mitigation strategies integrated into these systems; and (7) highlight cross-cutting limitations related to longitudinal use, inclusivity, diversity, implementation, and ethics. Table 1 summarizes the research questions guiding this review under four thematic categories: Detection, Evaluation, Mitigation, and Cross-cutting challenges.

**Table 1:** Categorized research questions guiding the scoping review.

Category	Research question	Purpose/focus
Detection	**RQ1.** What non-invasive technologies and modalities have been used to detect stress in children and adolescents, and what parameters do they assess?	Identify technology types (e.g., wearables, mobile systems, smart objects, contactless sensing) and the stress-related signals measured
	**RQ2.** How is stress induced and validated or evaluated in pediatric studies?	Examine stress induction approaches, task designs, contextual stressors, and ground-truth or validation methods
	**RQ3.** In what settings and age groups have these technologies been applied?	Explore deployment contexts (e.g., home, school, laboratory, therapy, clinic) and developmental stages
Evaluation	**RQ4.** What detection methods, frameworks, and evaluation approaches are used, and how accurate or effective are they?	Review statistical, machine learning, deep learning, and other analytic approaches, together with their evaluation metrics
	**RQ5.** What datasets are available for pediatric stress detection or evaluation, and what are their characteristics and collection methodologies?	Summarize dataset properties such as size, modality, annotation, study design, and accessibility
Mitigation	**RQ6.** What mitigation strategies or intervention frameworks have been integrated with these systems to reduce stress in children?	Identify biofeedback, adaptive support, serious games, smart objects, and caregiver- or parent-mediated intervention strategies
Cross-cutting	*(Discussion theme)* What limitations remain in relation to long-term monitoring, inclusivity, cultural diversity, implementation, and ethics in the current literature?	Highlight broader gaps affecting ecological validity, equity, translation, and responsible deployment

By addressing these questions, this review provides a structured synthesis of the current evidence base and outlines priorities for future research and practical implementation of non-invasive pediatric stress technologies.

## 2 Methodology

### 2.1 Review framework

This scoping review was guided by the Preferred Reporting Items for Systematic Reviews and Meta-Analyses extension for Scoping Reviews (PRISMA-ScR) [5]. A scoping review approach was selected because it enables systematic mapping of the existing literature, identification of key concepts, and clarification of research gaps without requiring exclusion of studies based on formal risk-of-bias appraisal, which aligns with the exploratory aims of this review [6].

### 2.2 Eligibility framework

The review question and eligibility criteria were structured using a PICO/PICOS-informed framework, consistent with systematic review reporting expectations. The population of interest was children and adolescents aged 0-18 years. The intervention or exposure of interest was any non-invasive digital or technological approach used for stress detection, monitoring, or mitigation. Comparators were not required for inclusion because the review included feasibility, observational, single-arm, and system-evaluation studies; however, studies with baseline, control, pre/post, stress/non-stress, or task-condition comparisons were eligible. Outcomes included stress detection, monitoring, mitigation, physiological or behavioral stress indicators, model performance, feasibility, usability, engagement, awareness, or intervention-related effects. Eligible study designs included peer-reviewed empirical journal articles and conference proceedings.

We use two distinct terms throughout this review. *Non-invasive* denotes methods that do not breach the body or require biological sampling, that is, no needles, blood draws, or salivary or cortisol collection in contrast to the invasive or burdensome conventional assessments described in the Introduction. *Non-contact* is a narrower property denoting no physical contact with the body, such as camera- or microphone-based sensing. Under these definitions, surface wearables (e.g., Empatica E4, Garmin, Shimmer) are *non-invasive but contact-based*, whereas audio- or vision-only systems are both non-invasive and non-contact. We treat these as two separate axes (Section 3.3, Table 2) and do not conflate them.

**Table 2:** Taxonomy used in this review: deployment form factor vs. sensing modality. Counts in Section 3.3 refer to form factor; counts in later sections may refer to sensing modality or evaluation strategy.

Axis	Categories	Examples
Deployment form factor	Wearable; App-based; Ambient/object-integrated; Hybrid	Wristbands, smartwatches, biosensor garments; mobile apps and dashboards; smart objects, robotic systems, ambient displays; multimodal wearable+app+visual systems
Sensing modality	Contact physiological; Non-contact; Hybrid/multimodal	HR/HRV, EDA, temperature, ACC; audio, facial image analysis; combined physiological, behavioral, contextual, or visual streams

For the age-based synthesis, the eligible population spanned 0–18 years inclusive, and studies were classified by their reported sample age range as follows: *Children* (samples wholly below 13 years), *Adolescents* (samples wholly within 13–18 years), *Mixed-age* (samples spanning both bands), and *Unclear/not reported*. The 13-year boundary was applied consistently, and assignment was based on the reported sample range rather than on individual participants.

No formal review protocol was registered for this scoping review. However, the review questions, eligibility criteria, search strategy, and extraction categories were defined before final screening and data charting.

### 2.3 Search strategy

We searched four bibliographic databases (PubMed, Scopus, IEEE Xplore, and ACM Digital Library) and screened Google Scholar as a supplementary source. Searches covered January 2013 to April 2026 (last search: April 2026). Database-specific strategies and exact queries are reported in Multimedia Appendix 2. The full database-specific search strings, including Boolean operators, field tags, date limits, and language filters, are provided in Multimedia Appendix 2.

- **Population**: “children,” “adolescents,” “pediatric,” “youth.”
- **Technology**: “non-invasive,” “wearable,” “ambient sensors,” “mobile applications,” “contactless,” “physiological monitoring,” “behavioral biometrics,” “ubiquitous computing.”
- **Outcome**: “stress,” “stress detection,” “stress monitoring,” “stress regulation,” “stress management,” “anxiety,” “emotion regulation.”

The search queries were adapted to each database’s syntax and indexing structure and were limited to English-language publications within the review time frame.

Google Scholar was used solely as a supplementary source to capture relevant records not indexed in the four primary databases. Due to the high volume of automated returns, results were sorted by default relevance, and a hard stopping rule was applied wherein only the first 200 records (20 pages) were screened. All records that passed this supplementary title/abstract screening were subjected to the same full-text eligibility assessment as the primary database records.

### 2.4 Inclusion and exclusion criteria

Studies eligible for inclusion met the following criteria:

- Focused explicitly on children and adolescents (0–18 years).
- Evaluated non-invasive technological methods for stress detection, monitoring, or mitigation.
- Reported empirical outcomes related to stress measurement, monitoring, or management.
- Were published as peer-reviewed journal articles or conference proceedings.

Studies were excluded if they:

- Targeted adult populations exclusively.
- Used invasive or physically intrusive methodologies.
- Were purely theoretical, non-empirical, or lacked original empirical or system-evaluation outcomes.
- Were literature reviews, editorial commentaries, opinion pieces, dissertations, theses, or other non-peer-reviewed publications.

### 2.5 Study selection process

The study selection process followed three phases: identification, screening, and eligibility assessment. Search results were exported into Zotero for duplicate removal and screening management. After duplicates were removed, title and abstract screening was conducted to determine whether studies met the core inclusion criteria: (1) relevance to stress, (2) relevance to children or adolescents, and (3) use of non-invasive technological approaches. Studies that appeared eligible on the basis of title and abstract were then assessed at full-text level.

Full texts of potentially eligible articles were reviewed against the predefined inclusion and exclusion criteria. Studies meeting all criteria were retained for data charting and synthesis. Disagreements during screening and eligibility assessment were resolved through discussion among the review team.

Three reviewers participated in study selection. One reviewer conducted the primary title and abstract screening and full-text eligibility assessment for the entire dataset. The second and third reviewers each independently screened and verified 50% of the records. Any disagreements were resolved through discussion and consensus among all three reviewers. Reviewers were not blinded to authors, institutions, or publication venues; blinding is not required for scoping reviews under PRISMA-ScR, and the exploratory mapping aim of the review does not rely on effect-size estimation where reviewer blinding is relevant.

### 2.6 Data extraction and analysis

We used both deductive and inductive approaches to chart relevant study characteristics [7, 8]. Initially, data categories were predefined based on prior literature and domain knowledge relevant to non-invasive stress technologies [9, 10]. Using this deductive framework, we extracted information on sensing technologies, physiological signals (eg, heart rate variability, electrodermal activity, skin temperature, respiration), behavioral indicators (eg, facial activity, speech, gaze, movement), and device form factors such as wristbands, smartwatches, garments, smart objects, or contactless sensing systems [11].

We also extracted information on hardware and software setup where reported, as well as interaction modalities used for presenting stress-related information, including visual, auditory, and tactile feedback. Contextual variables included study setting (e.g., school, home, laboratory, therapy, clinic), participant characteristics (e.g., age group, neurodiversity, participant role), experimental conditions, stress induction methods, and ground-truth or validation measures. In addition, we charted the main findings reported by authors regarding system feasibility, performance, usability, stress awareness, monitoring capability, or mitigation value.

One reviewer led data extraction using a charting form in Google Sheets. Extracted data and uncertain cases were checked and discussed with the second and third reviewers. The charting form captured: study design and setting; participant characteristics; sensing modality and form factor; stress induction and validation; modeling and evaluation approach; datasets and dataset availability; and mitigation or intervention features where present. Data charting followed PRISMA-ScR recommendations for iterative refinement of the extraction framework. The complete per-study extraction table is provided in Multimedia Appendix 1.

### 2.7 Data synthesis

Extracted data were synthesized descriptively and thematically. First, study characteristics were summarized using frequencies and narrative descriptions, including publication year, setting, age group, sensing modality, study design, dataset availability, and mitigation features. Second, studies were grouped thematically according to the review questions: technologies and modalities, stress induction and validation methods, study contexts and age groups, evaluation approaches, datasets, mitigation strategies, and cross-cutting implementation challenges.

For the synthesis of stress induction methods, each study was assigned to one primary stressor category based on the dominant mechanism through which stress was elicited or monitored. *Cognitive/Academic* refers to learning-, homework-, exam-, or performance-related task demands; *Social/Evaluative* refers to stress arising from observation, judgment, interview-like situations, or socially demanding interaction; *Game/Interactive* refers to stress elicited within serious games, interactive digital tasks, or play-based challenge environments; *Naturalistic/Passive* refers to studies that monitored stress as it emerged during everyday activities without imposing a formal experimental stressor; and *Clinical/Procedural* refers to stress associated with treatment procedures, clinical workflows, or healthcare encounters. Where studies could plausibly fit more than one category, assignment was based on the primary stress context emphasized by the study design.

Because of heterogeneity in study design, participant groups, stress labels, sensing modalities, and reported outcomes, meta-analysis was not appropriate. Findings were therefore synthesized narratively, with tables used to support cross-study comparison.

### 2.8 Quality assessment and risk of bias

Although formal risk-of-bias assessment is not mandatory for scoping reviews, we conducted a lightweight methodological quality appraisal to align the review with systematic review reporting expectations and to help interpret the strength of the included evidence. The purpose of this appraisal was not to exclude studies, but to contextualize findings across heterogeneous study designs.

Each included study was assessed using a pragmatic quality-appraisal framework adapted for pediatric technology studies. We considered five domains: (1) clarity of study aim and target population; (2) appropriateness of the study design for evaluating stress detection, monitoring, or mitigation; (3) adequacy of stress induction, labeling, or validation procedures; (4) clarity of sensing, intervention, or system description; and (5) completeness of outcome reporting, including performance, feasibility, usability, or intervention-related outcomes where applicable.

Studies were categorized as having lower, moderate, or higher methodological concern based on the number and severity of limitations identified across these domains. Studies with clearly defined samples, transparent validation procedures, and complete outcome reporting were considered to have lower methodological concern. Studies with partially described validation procedures, small or narrowly defined samples, or limited reporting of technical or evaluation details were considered to have moderate concern. Studies with unclear validation logic, minimal empirical evaluation, or incomplete reporting of participant, system, or outcome details were considered to have higher concern.

Quality appraisal was used narratively during synthesis and did not determine inclusion or exclusion. This approach was selected because the included studies varied substantially in design, including feasibility studies, observational studies, machine-learning evaluations, intervention studies, and field deployments. The per-study appraisal ratings (lower, moderate, or higher methodological concern) across these five domains are reported in Supplementary Material S4.

### 2.9 Study selection

**Figure 1:**
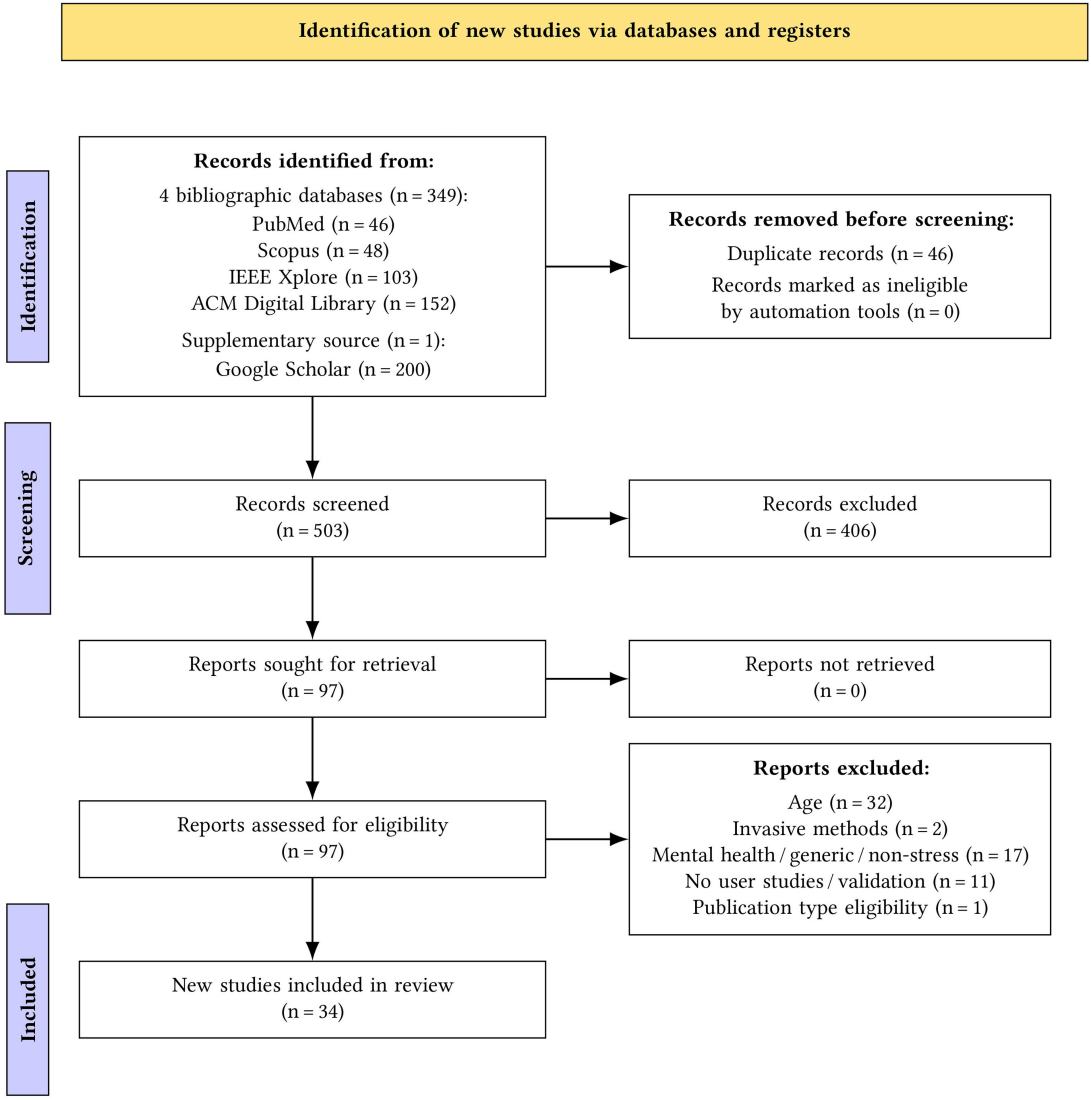
PRISMA-ScR flow of study selection.

Our search identified 549 records across all sources: PubMed (*n* = 46), Scopus (*n* = 48), IEEE Xplore (*n* = 103), ACM Digital Library (*n* = 152), and Google Scholar supplementary screening (*n* = 200). After removing duplicates (*n* = 46), 503 records remained for title and abstract screening. Of these, 406 records were excluded. We then assessed 97 full-text reports for eligibility and excluded 63 reports with reasons, including adult-only samples, invasive methods, lack of stress-specific outcomes, non-empirical designs, or publication types outside the eligibility criteria. A total of 34 studies met the inclusion criteria and were retained for data charting and synthesis. The PRISMA-ScR flow is shown in Figure 1.

## 3 Results

A total of **34 studies** were included in this review (Figure 1).

### 3.1 Publication characteristics

**Figure 2:**
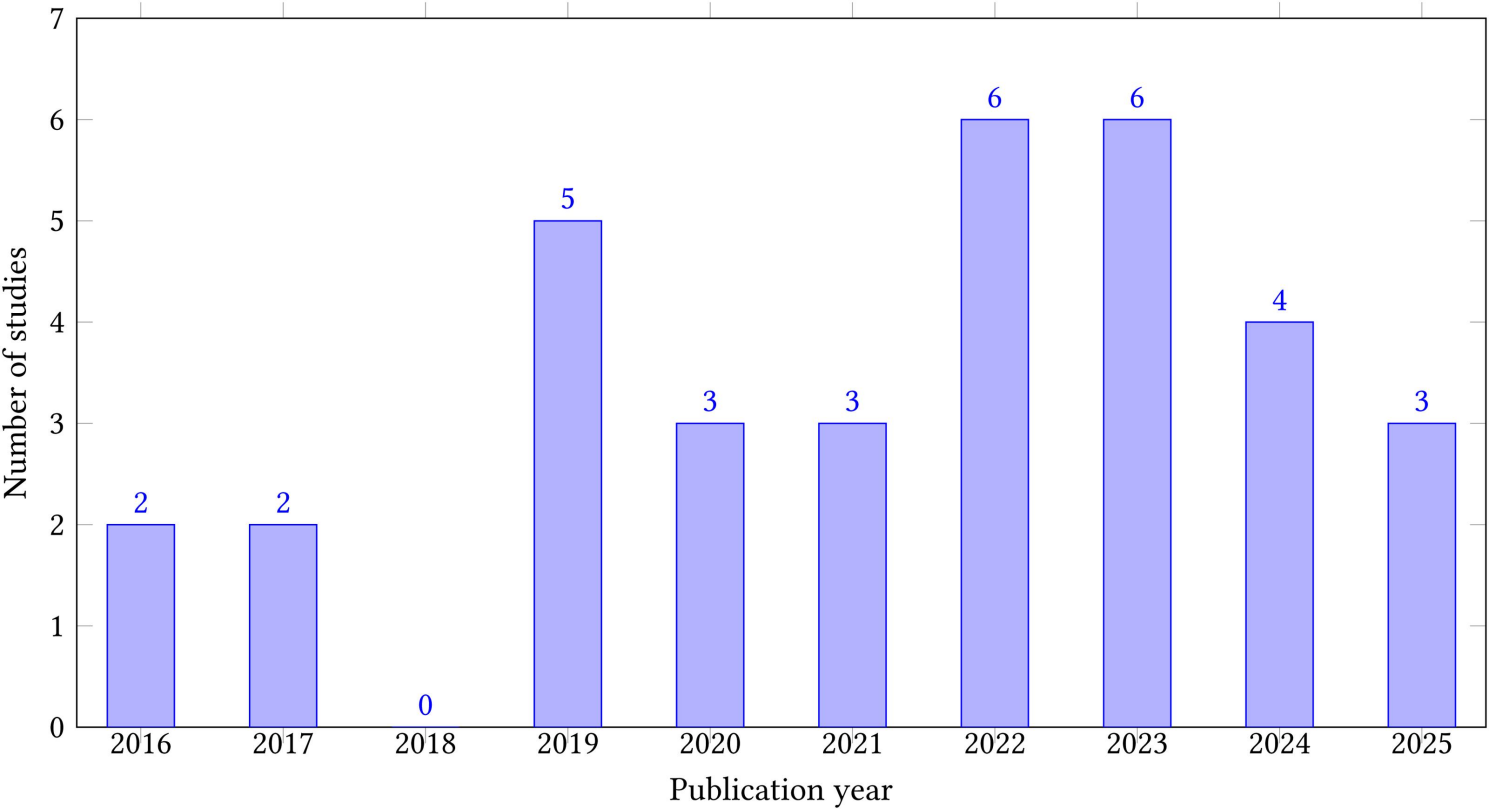
Distribution of included studies by publication year (n=34).

A total of 34 studies were included in the review. Based on the extracted data, the included studies were published between 2016 and 2025. Publication activity was highest in 2022 (6 studies) and 2023 (6 studies), followed by 2019 (5 studies) and 2024 (4 studies). The years 2020, 2021, and 2025 each contributed 3 studies, while 2016 and 2017 contributed 2 studies each. Overall, the distribution indicates sustained growth in pediatric non-invasive stress research, particularly from 2019 onward (Figure 2).

### 3.2 Methodological quality of included studies

The methodological quality appraisal indicated variation in reporting completeness and evaluation rigor across the included studies. Studies with lower methodological concern generally provided clear participant descriptions, detailed sensing or intervention procedures, explicit stress labeling or validation methods, and complete outcome reporting. Studies with moderate concern reported empirical outcomes but often had limitations such as small samples, limited detail on label generation, short deployment duration, or incomplete model validation reporting. Studies with higher concern were typically early-stage feasibility or prototype evaluations with limited validation detail.

No studies were excluded on the basis of methodological quality. Instead, appraisal findings were used narratively to interpret the maturity of the evidence base and to contextualize limitations related to small samples, heterogeneous validation methods, and limited dataset openness.

### 3.3 Technologies and modalities used

This section categorizes the technological approaches used for stress monitoring, detection, and mitigation in children and adolescents across the 34 included studies. We analyze technologies along two complementary axes: (1) *deployment form factor*, which describes how a system is delivered (e.g., wearable, app-based, ambient/object-integrated, or hybrid), and (2) *sensing modality*, which describes how signals are acquired (e.g., contact-based physiological sensing, non-contact sensing, or multimodal/hybrid sensing).

Based on the extracted data, the included systems can be broadly described as **wearable-dominant** (**23/34**), **app-only** (**3/34**), **ambient or object-integrated** (**5/34**), and **explicit hybrid systems** (**3/34**). At the sensing level, most studies relied primarily on **contact-based physiological signals**, while a smaller subset used **audio**, **vision**, or multimodal combinations.

#### 3.3.1 Wearable systems

Wearable technologies formed the dominant deployment pattern in the dataset. These systems included wristbands, smartwatches, wearable biosensor suites, wearable feedback devices, and wearable systems paired with mobile or therapeutic platforms. Physiological signals such as heart rate (HR), electrodermal activity (EDA), heart rate variability (HRV), skin temperature, and accelerometry were the most common measures (Table 3).

**Table 3:** Representative wearable-based stress monitoring systems.

Authors (Year)	Device/system	Modality	Parameters Captured
Aktas et al. (2022) [12]	Empatica E4 sensor suite	Physiological	EDA, HR, ST, ACC
Antle et al. (2019) [22]	Biofeedback game with wearable sensors	Physiological + Behavioral	HR, EDA, interaction data
Choi et al. (2017) [23]	Wrist-worn biosensors	Physiological	GSR, HR, temperature
Deng et al. (2022) [13]	Wearable sensing + mobile logging	Physiological + Contextual	HR, temperature, EDA, motion
Jin et al. (2023) [14]	Wearable device data for stress prediction	Physiological	HRV and wearable-derived features
Northrup et al. [19] (2016)	Wearable stress sensors with caregiver alerts	Physiological	Physiological stress traces + alert triggers
Ludovichetti et al. (2025) [17]	Garmin-based wearable monitoring	Physiological	HRV-derived stress score
Jaldin et al. [18] (2025)	Shimmer3 GSR+ system	Physiological + Motion	GSR, PPG-derived HR, hand movement

Examples include Empatica-based sensing in Aktas et al. [12] and Deng et al. [13], smartwatch or wrist-worn sensing in Jin et al. [14], Nguyen et al. [15], Yu et al. [16], and the dental monitoring studies by Ludovichetti et al. [17] and Jaldin et al. [18]. Wearables were also used in autism-focused intervention contexts, including Northrup et al. [19], Masino et al. [20], and the Coşkun [21] studies.

#### 3.3.2 Ambient and object-integrated systems

Ambient and object-integrated systems were less common in the included studies but remained conceptually important. These included classroom or home-based smart objects, peripheral feedback devices, smart toys, and robotic platforms. This category included studies such as Chyan et al. [24], Theofanopoulou et al. [25], Yao et al. [26], and robot-mediated systems such as Kumazaki et al. [27] (Table 4).

**Table 4:** Representative ambient or object-integrated systems.

Authors (Year)	System	Modality	Parameters captured
Chyan et al. [24] (2023)	Multi-speaker audio stress detection setup	Audio	Speech pitch, MFCC, prosody, energy
Kumazaki et al. [27] (2017)	Android robot intervention	Behavioral	Observer-coded interaction behavior
Theofanopoulou et al. [25] (2019)	Smart toy for emotion regulation	Behavioral + Interaction	Toy interaction metrics, child behavior
Yao et al. [26] (2023)	StressButton smart object	Behavioral	Coping-trigger interactions and usage context
Chaowadee et al. [28] (2021)	Stress warning unit in classroom learning context	Physiological + Object feedback	HR, GSR, temperature

#### 3.3.3 Multimodal and hybrid systems

A smaller but important subset of studies integrated multiple sensing or delivery channels. These hybrid systems combined physiological data with behavioral, contextual, visual, audio, or interface-based components. Examples include Zhang et al. [29], which used multimodal fusion; Li et al. [30], which combined wearable biofeedback with an ambient diffuser and parent-facing interface; Thierfelder et al. [31], which used multimodal sensor fusion; and Loftness et al. [32], which explicitly optimized multimodal wearable instrumentation (Table 5).

**Table 5:** Representative multimodal/hybrid systems.

Authors (Year)	System	Modality	Parameters captured
Zhang et al. [29] (2020)	Multi-modal interactive fusion model	Multimodal	Text, image, sleep, exercise, contextual data
Li et al. [30] (2024)	Stress Diffuser biofeedback system	Wearable + Ambient	PPG/HRV-derived stress, contextual interaction
Loftness et al. [32] (2022)	UVM KID multimodal stress study	Physiological + Visual + Audio	Physiological, visual, and audio features
Thierfelder et al. [31] (2022)	Multimodal sensor-based identification system	Physiological + Audio + Motion	Sensor fusion for stress/compulsive action identification
Vural et al. [33] (2024)	Facial-image stress recognition during physiotherapy games	Visual	Facial stress patterns during game-based physiotherapy
Yu et al. (2023) [16]	Smartwatch-based feasibility system with caregiver tagging	Physiological + Behavioral	Physiological data with caregiver-informed contextual tagging

Across the included studies, physiological sensing remained the dominant technical foundation, especially through wearable platforms. Non-contact sensing approaches were comparatively rare, and explicit multimodal fusion systems remained limited. To make these counts explicit and reproducible, we classified systems by sensing modality using the following criteria: *purely non-contact* systems acquire all stress-relevant signals without body contact (audio-only or vision-only); *hybrid/multimodal* systems combine at least one non-contact channel with other streams; and all remaining systems are *contact-based physiological*. On this basis, the corpus contained 2 purely non-contact systems [audio-only: Chyan et al. [24]; vision-only: Vural et al. [33]] and 3 hybrid/multimodal systems incorporating a non-contact channel [Loftness et al. [32], Thierfelder et al. [31], and Zhang et al. [29]]; the remaining systems were contact-based. Nevertheless, the review corpus includes a broad spread of deployment types, including school-facing smart objects, toy-based interventions, caregiver-linked home systems, and clinical procedural monitoring in dentistry.

### 3.4 Stress induction and validation methods

Understanding how stress is induced and validated in pediatric populations is central to evaluating the effectiveness and ecological relevance of non-invasive stress technologies. In the set of 34 included studies, 32 studies reported either an explicit stress induction protocol or monitoring under identifiable naturalistic, contextual, or procedural conditions. Two studies were better characterized as dataset resources or early-stage empirical evaluations without a fully described induction-validation protocol.

#### 3.4.1 Stress induction modalities

In the included studies, stress-related conditions clustered into five broad groups: cognitive/academic, social/evaluative, game-based/interactive, naturalistic/passive, and clinical/procedural. This grouping reflects the extracted data, including courtroom and dental-procedure studies.

Cognitive and academic stressors included learning tasks, timed visual tasks, homework, schoolwork, and exam-related pressure. These were especially common in school-linked or home-learning studies. Social and evaluative stressors were used in mock interviews, courtroom testimony, socially demanding therapeutic sessions, and robot-mediated interaction contexts. Game-based and interactive paradigms remained important for child-centered elicitation, especially in autism, physiotherapy, or gamified self-regulation research. Naturalistic and passive studies monitored stress as it emerged in daily routines, classrooms, homes, or therapy environments. Finally, the dataset also included clinical/procedural stressors, especially in pediatric dentistry, where stress was monitored across treatment phases or invasive vs. non-invasive procedures.

#### 3.4.2 Validation methods

Validation approaches in the dataset were heterogeneous. Physiological validation remained common, particularly through HRV, HR, EDA, ECG, temperature, and signal-threshold approaches. Self-report tools were also widely used, especially in older children and adolescents, often through numeric scales, stress questionnaires, or reflective journaling. Observational or expert-labeled approaches were frequently used in autism, therapy, or clinical contexts, including therapist coding, caregiver tagging, clinician diaries, and video annotation. Mixed validation approaches, combining physiological and observational or self-report evidence, were also common.

#### 3.4.3 Structure of the evidence

For synthesis, the studies are organized below into five groups: cognitive/academic, social/evaluative, game-based/interactive, naturalistic/passive, and clinical/procedural (Table 6).

**Table 6:** Included studies grouped by primary stressor category, with induction method, validation method, signals or instruments, and study context. Studies are assigned to a single dominant stressor category as defined in Section 2.7.

Author(s) (Year)	Induction method	Validation method	Signals/instruments	Context used
Cognitive/Academic				
Chaowadee et al. [28] (2021)	Learning tasks with escalating difficulty	Thresholds + student-reported experience	Physiological	School
Choi et al. [23] (2017)	Timed visual tasks	Self-report post-task	Physiological	School
Jin et al. [14] (2023)	Academic exam periods (naturalistic)	Self-report questionnaires	Physiological	School
Li et al. [30] (2024)	Homework task with parent involvement	EDA thresholds + observational cues	Physiological + Contextual	Home
Yao et al. [26] (2023)	Homework/learning stress (natural)	Self-report and use context	Behavioral	Home + school
Zhang et al. [29] (2020)	Natural academic/social pressure	User feedback on app	Multimodal contextual data	Home + school
Social/Evaluative/Robot-mediated				
Aktas et al. [12] (2022)	Therapy session with predefined stress triggers	Expert observation + video annotation	Physiological	Therapy center
Chyan et al. [24] (2023)	Emotionally charged prompts and scenarios	Self-report + observer annotation	Audio	School environment
Coşkun et al. [34] (2022)	Robot-based interactions with increasing complexity	Observer coding + EDA thresholds	Physiological + Behavioral	Therapy room
Kumazaki et al. [27] (2017)	Social scenarios with robot	Observer ratings + video coding	Behavioral	Clinic
Loftness et al. [32] (2022)	School tasks + mock interviews	Observer ratings + PSS-C	Physiological, visual, audio	School
Rodriguez-Pellejero et al. [35] (2024)	Witnessing/legal testimony	Context-based court-session phase	Wearable physiological sensing	Courtroom
Sarabadani et al. [36] (2020)	Behavior sessions with social/attention triggers	Video-coded behavioral categories	Physiological	Clinical
Game-based/Interactive				
Antle et al. [22] (2019)	Gameplay challenges and narrative stressors	Pre/post self-report + session logs	Physiological + Behavioral	School
Carlier et al. [37] (2019)	Mild challenges within game environment	Adapted self-report	Behavioral	Therapeutic center
Coşkun et al. [21] (2023)	Timed and competitive game scenarios	Observer + self-report	Physiological	Therapeutic center
Kim et al. [38] (2021)	Educational games with increasing difficulty	Self-report + game performance	Physiological	School
Nicolaidou et al. [39] (2019)	Stress scenarios within game/app use	Self-reported stress scales	Behavioral/self-report	School
Theofanopoulou et al. [25] (2019)	Performance-linked interactive play/toy use	Self-report + observed behavior	Behavioral + interaction	Mixed setting
Vural et al. [33] (2024)	Physiotherapy game activity	Video annotation by therapists	Visual	Clinic + home
Naturalistic/Passive				
Curzio et al. [40] (2022)	Baseline stress before horticultural therapy	PSS + salivary cortisol	Physiological + psychological	Hospital therapy garden
Masino et al. [20] (2019)	Naturalistic school routines	Teacher and therapist annotations	Physiological	School
Nguyen et al. [15] (2021)	Daily life (in-situ)	Self-reported awareness + journaling	Physiological	Home
Northrup et al. [19] (2016)	Daily classroom and therapy activities	Caregiver observation matched to sensor data	Physiological	School + therapy
Redd et al. [41] (2020)	Naturalistic home and clinic monitoring	Clinician rating + behavioral diaries	Physiological	Home + clinic
Wang et al. [42] (2025)	Naturalistic home setting	Parental observations + reflection	Behavioral	Home
Yu et al. [16] (2023)	Naturalistic daily-life monitoring	Caregiver-tagged stress episodes	Physiological + Behavioral	Home
Clinical/Procedural				
Ludovichetti et al. [17] (2025)	Dental procedures across treatment phases	Event-based phase labeling	Wearable HRV-based monitoring	Pediatric dentistry clinic
Jaldin et al. [18] (2025)	Invasivevs. non-invasive dental treatment	Protocol-timed readings + self-report + dentist ratings	GSR, PPG/HR, motion	Public dental clinic

Two included studies were not easily classifiable within the stressor categories above: the observational database paper by Coşkun et al. [43], which focused on dataset construction and therapist annotation, and the early-stage wearable study by Gul Airij et al. [44], which reported empirical or system-evaluation outcomes but did not include a fully described induction-validation protocol.

Overall, the included studies show substantial heterogeneity in how stress was elicited or monitored. Standardized pediatric protocols remained rare, and validation strategies varied widely across self-report, observation, physiological thresholds, and mixed approaches.

### 3.5 Study contexts and age groups

This section synthesizes the deployment contexts and age groups targeted by the 34 included studies. These dimensions are central to understanding ecological validity, developmental fit, and translational potential.

The included studies were not limited to laboratory-only settings. Instead, the corpus is distributed across school-based settings (9 studies), clinical/therapy settings (9 studies), mixed settings (7 studies), home-only settings (4 studies), other/unspecified settings (4 studies), and one courtroom study (Table 7). This distribution indicates a broader shift toward ecologically relevant and applied settings, even though purely home-based and longitudinal work remains limited.

**Table 7:** Distribution of studies by environment type (*n* = 34 total).

Environment type	Number of studies	Representative examples
School-based	9	Antle et al. [22]; Chaowadee et al. [28]; Choi et al. [23]; Jin et al. [14]; Loftness et al. [32]
Clinical/therapy	9	Aktas et al. [12]; Carlier et al. [37]; Kumazaki et al. [27]; Sarabadani et al. [36]; Ludovichetti et al. [17]; Jaldin et al. [18]
Mixed-context	7	Coşkun et al. [43]; Zhang et al. [29]; Vural et al. [33]; Northrup et al. [19]; Yao et al. [26]
Home-only	4	Li et al. [30]; Nguyen et al. [15]; Wang et al. [42]; Yu et al. [16]
Other/unspecified	4	Gul Airij et al. [44]; Melinda et al. [45]; Theofanopoulou et al. [25]
Courtroom	1	Rodriguez-Pellejero et al. [35]

**Figure 3:**
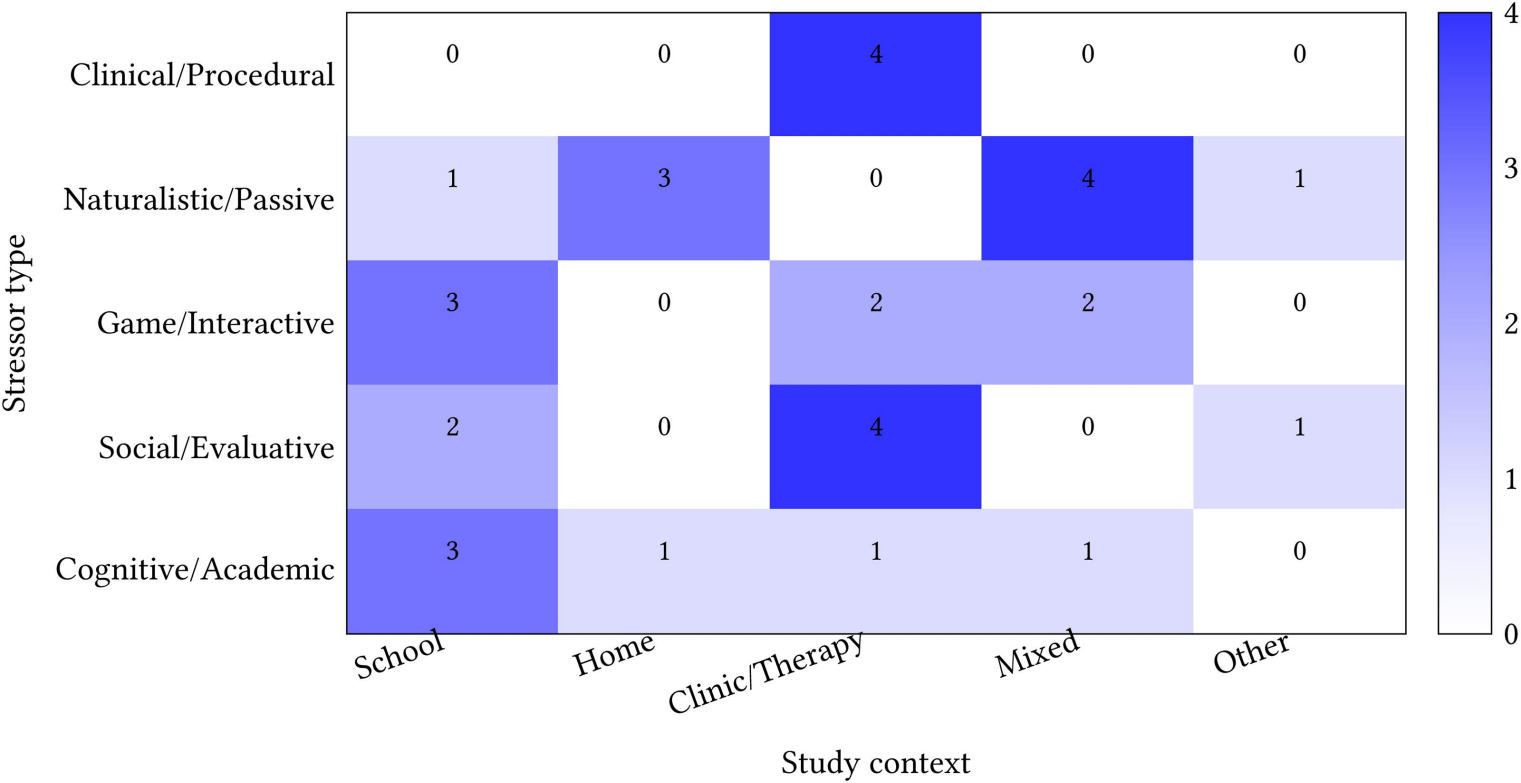
Heatmap of primary study context by primary stressor type across the included studies. Each study was assigned to one dominant context and one dominant stressor category. One study without a sufficiently specified empirical stressor-context structure was excluded from this visualization, resulting in n=33.

School-based studies remained prominent, particularly for learning-related stress, physiological monitoring, and classroom-friendly interventions (Figure 3).

#### 3.5.1 Targeted age groups

Using the extracted data, children under 13 years dominated the review corpus (25/34, 73.5%). Only 3 studies focused exclusively on adolescents aged 13–18 years (8.8%), while 5 studies included mixed-age samples (14.7%) and 1 study did not clearly specify age (2.9%) (Table 8). Thus, the literature is more heavily concentrated on children than on adolescents.

**Table 8:** Distribution of studies by age group (*n* = 34 total).

Age group	Number of studies	Representative examples
Children (<13 years)	25	Antle et al. [22]; Carlier et al. [37]; Choi et al. [23]; Li et al. [30]; Theofanopoulou et al. [25]; Yu et al. [16]
Adolescents (13–18 years)	3	Curzio et al. [40]; Jin et al. [14]; Jaldin et al. [18]
Mixed-age samples	5	Aktas et al. [12]; Kumazaki et al. [27]; Nguyen et al. [15]; Rodriguez-Pellejero et al. [35]; Zhang et al. [29]
Unclear/not reported	1	Gul Airij et al. [44]

#### 3.5.2 Demographic representation

Neurodivergent populations, especially children with autism spectrum disorder (ASD), were strongly represented in the included studies. Based on the extracted data, 12 of 34 studies (35.3%) explicitly focused on ASD or closely related autism-linked stress contexts. This reflects both the perceived need for non-invasive stress monitoring in neurodivergent groups and the feasibility of wearable or interactive systems in autism-related research. At the same time, broader demographic diversity including ethnicity, socioeconomic variability, and representation beyond ASD-focused cohorts, was still inconsistently reported across the review corpus.

Overall, the evidence base is clinically and school-context grounded, and it is substantially more child-focused than adolescent-focused. Purely home-based work and long-term in-the-wild deployment remain limited despite their importance for ecological validity.

### 3.6 Evaluation approaches and performance of detection models

The reviewed studies used diverse evaluation strategies, ranging from quantitative classification and statistical comparison to mixed-methods feasibility studies, qualitative field evaluation, and early-stage empirical feasibility work. Based on the extracted data, the evaluation landscape can be summarized as follows: 20 studies were predominantly quantitative, 6 studies used mixed or hybrid evaluation, 5 studies were primarily qualitative or field-based, and 3 studies were best described as early-stage empirical or feasibility-oriented (Table 9).

**Table 9:** Evaluation approaches and representative outcomes in the 34-study set.

Evaluation method	Number of studies	Typical reported outputs	Representative examples
Predominantly quantitative	20	Accuracy, F1-score, precision, significance tests, repeated-measures comparisons	Aktas et al. [12]; Choi et al. [23]; Vural et al. [33]; Zhang et al. [29]; Jin et al. [14]; Sarabadani et al. [36]; Ludovichetti et al. [17]
Mixed/hybrid evaluation	6	Combined performance metrics + interviews, logs, or user feedback	Antle et al. [22]; Carlier et al. [37]; Nicolaidou et al. [39]; Redd et al. [41]; Theofanopoulou et al. [25]; Yu et al. [16]
Qualitative/field-based	5	Feasibility, usability, engagement, reflection, child-caregiver interpretation	Coşkun et al.[43] (dataset-focused paper); Nguyen et al. [15]; Northrup et al. [19]; Wang et al. [42]; Yao et al. [26]
Early-stage empirical/feasibility-oriented	3	Pilot evaluation, proof-of-concept implementation, feasibility outcomes, or unbenchmarked rule-based system evaluation	Gul Airij et al. [44]; Li et al. [30]; Melinda et al. [45]

#### 3.6.1 Quantitative and statistical evaluations

Quantitative evaluations remained the dominant approach in the review corpus. These studies used statistical comparison, confusion matrices, cross-validation, accuracy, F1-score, precision, significance testing, or repeated-measures analysis. Reported performance metrics varied by modality and task. In the extracted data, several machine-learning or statistical studies reported performance in the broad range of F1 ≈ 0.79–0.91 and accuracy ≈ 82%–92%, although direct comparison is limited by substantial variation in sample size, context, target labels, and outcome definition.

#### 3.6.2 Machine learning and deep learning

Machine-learning approaches were present in multiple studies, particularly in wearable physiological classification work. Based on the extracted data, supervised machine-learning methods (e.g., SVM, Random Forest, kNN, logistic regression, or multimodal fusion models) appeared in at least 9 studies. Deep learning was used explicitly in 3 studies: Chyan et al. [24], Vural et al. [33], and Jin et al. [14]. The highest clearly charted classification performance in the extracted data was approximately 92%, reported in studies such as Zhang et al. and Vural et al. Chyan et al. reported 88.4% accuracy for speech-based deep learning in a noisy school setting.

#### 3.6.3 Mixed and qualitative evaluations

Mixed-methods studies were especially important for child-centered interventions, serious games, smart toys, and caregiver-facing systems. These studies combined behavioral logs, interviews, observations, self-report, usability findings, or pre/post emotional measures. Qualitative studies contributed insight into feasibility, engagement, caregiver interpretation, and child acceptability, especially in home, school, and therapeutic contexts.

#### 3.6.4 Early-stage empirical and feasibility-oriented work

A small number of studies were better interpreted as early-stage empirical or feasibility-oriented evaluations. These included pilot systems, prototype evaluations, and feasibility studies that reported empirical outcomes but did not provide strong benchmarked classification performance. They remain useful for design-space expansion but should not be interpreted as equivalent to validated predictive systems.

Overall, the included studies suggest that pediatric stress technology research remains dominated by quantitative and small-sample empirical evaluation, with a smaller but important body of mixed and qualitative work focused on feasibility, adoption, and regulation support. The field continues to face trade-offs among methodological rigor, ecological validity, sample size, and developmental appropriateness.

### 3.7 Stress datasets and data collection methodologies

Datasets remain foundational to pediatric stress technology research because they support benchmarking, model development, and replication. Based on the extracted data, 31 of the 34 studies involved some form of original or study-specific dataset generation or structured data collection, whereas 3 studies were marked as not dataset-based or not applicable.

#### 3.7.1 Types and availability of datasets

Most studies relied on custom or study-specific datasets. In the extracted data, 28 studies were marked as private/closed, 3 as public or partially public, and 3 as not applicable. This confirms that openly reusable pediatric stress datasets remain rare. The only clearly open dataset named in the extracted data was AKTIVES, while two Coşkun-related entries were marked as partially public or shared.

#### 3.7.2 Data modalities captured

Physiological sensing dominated the dataset landscape. Common modalities included HR, HRV, EDA, temperature, accelerometry, ECG, and related biosignals. A smaller subset incorporated audio, visual, behavioral, contextual, or multimodal data. In the included studies, most datasets were grounded in contact-based physiological sensing, with fewer studies using speech, facial image analysis, or broader contextual fusion.

#### 3.7.3 Labeling and annotation approaches

Three broad labeling approaches appeared repeatedly in the included studies:

(1) **Self-report-based labeling**, especially in school-aged children, adolescents, homework settings, and game-based studies;
(2) **Observer, therapist, caregiver, or clinician annotation**, especially in autism, therapy, and clinical environments; and
(3) **Event- or phase-based labeling**, where stress was inferred from task timing, procedural phases, or known contextual transitions, such as courtroom testimony or dental treatment stages.

#### 3.7.4 Population and sample characteristics

Based on the extracted data, reported sample sizes ranged from approximately 5 to 80 participants, with a median near 20. Many datasets were relatively small and context-specific. Neurodivergent populations, especially ASD-focused cohorts, were strongly represented in dataset creation, while large, demographically broad, publicly reusable datasets remained absent.

#### 3.7.5 Duration and context of data collection

Most data collection was conducted in single-session, short-term, or limited-duration studies. Longitudinal collection was relatively uncommon, although some studies monitored stress across repeated daily-life, school, or home contexts. Overall, school, clinical/therapy, and mixed settings were more common than purely home-based longitudinal monitoring.

In summary, the dataset analysis confirms that pediatric stress research still depends heavily on small, closed, study-specific datasets with heterogeneous annotation practices. This remains a major barrier to reproducibility, cross-study comparison, and more robust model development.

### 3.8 Mitigation strategies and interventions

While many systems in the review corpus focused primarily on detection or monitoring, the extracted data show that mitigation and intervention were also prominent. Specifically, 17 of the 34 studies included some form of mitigation, adaptive support, intervention, or caregiver-facing response component, whereas the remaining 17 studies were primarily detection- or monitoring-oriented (Table 10).

**Table 10:** Representative mitigation and intervention studies (selected examples rather than exhaustive list).

Study	Mitigation type	Signals used	Real-time or offline	Reported impact
Antle et al. [22] (2019)	Interactive biofeedback system	HR, EDA	Real-time/session-based	Improved self-regulation processes
Carlier et al. [37] (2019)	Serious game-based relaxation support	Behavioral + self-report	Session-based	Reduced anxiety and improved engagement
Chaowadee et al. [28] (2021)	Stress alerts for student and teacher	HR, GSR, temperature	Real-time	Improved learning accuracy and fewer stress episodes
Li et al. [30] (2024)	Biofeedback prompts with parent involvement	HRV-related biofeedback	Real-time	Improved stress awareness and parent-child interaction
Nguyen et al. [15] (2021)	Haptic alerts and reflection prompts	Physiological	Real-time	Increased anxiety/stress awareness
Theofanopoulou et al. [25] (2019)	Smart toy-based calming support	Interaction-based feedback	Near real-time	Improved self-soothing and regulation
Wang et al. [42] (2025)	Parent-facing reflective prompts	Behavioral/contextual	Near real-time	Better parent-child stress management dialogue
Yao et al. [26] (2023)	Child-initiated coping support via object interaction	Behavioral	Real-time/on-demand	High engagement and usable coping support
Yu et al. [16] (2023)	Caregiver-informed support based on smartwatch data	Physiological + contextual	Near real-time	Feasibility and improved caregiver awareness

#### 3.8.1 Types of mitigation strategies

Mitigation strategies in the included studies fell into several recurring forms:

(1) **Biofeedback and awareness support**, including stress displays, feedback loops, and calming prompts;
(2) **Game-based or toy-based regulation tools**, especially in child-centered and autism-related contexts;
(3) **Caregiver- or parent-facing support**, where monitoring data informed adult intervention or reflective prompting; and
(4) **Context-aware adaptive systems**, where feedback or system behavior changed in response to stress signals.

#### 3.8.2 Signals and modalities used

Most mitigation systems relied on physiological signals such as HR, HRV, EDA, or wearable-derived stress indicators. A smaller subset used behavioral interaction traces, speech, or contextual information. In the included studies, mitigation was not limited to wrist-worn biofeedback; it also appeared in serious games, smart toys, connected objects, caregiver dashboards, and adaptive home-learning systems.

#### 3.8.3 Real-time vs. offline adaptation

Both real-time and session-based mitigation appeared in the review corpus. Real-time or near-real-time support was especially visible in biofeedback, alerting, smartwatch, and parent-child support systems. Other interventions, such as serious games or post-session reflection tools, were more structured around sessions rather than continuous adaptation.

#### 3.8.4 Reported impacts

Most mitigation-oriented studies reported improvements in one or more of the following: self-awareness, emotional regulation, caregiver awareness, engagement, or reduced anxiety/stress indicators. However, these studies were generally short-term and context-specific. Longitudinal evidence on sustained benefit remains limited.

Taken together, the included studies suggest that mitigation is no longer a minor fringe category. Across the included studies, a substantial subset of the literature incorporated adaptive or supportive elements. However, truly scalable, longitudinal, age-personalized, and ethically robust intervention systems remain limited, especially outside school and therapy contexts.

## 4 Discussion

The 34 included studies reveal a field in active transition: meaningful progress in sensing design, child-centered intervention formats, and context-specific deployment, set against persistent methodological fragmentation in modality choice, stress induction logic, validation strategy, and outcome reporting. As introduced in Section 1, pediatric stress technologies carry developmental and public health significance beyond technical performance enabling timely, low-burden monitoring offers a pathway to prevent long-term cognitive and social consequences [1–3], and their integration into schools and homes represents a pedagogical and clinical priority, not merely an engineering one. The following subsections interpret the patterns identified in the Results across five thematic areas.

### 4.1 Non-contact methods in pediatric stress detection

This review confirms that pediatric stress research remains strongly dominated by contact-based sensing, particularly wearable systems using heart rate, heart rate variability, electrodermal activity, skin temperature, and motion signals. Wrist-worn or wearable-integrated systems were the dominant pattern across the review, reflecting the continued dependence of pediatric stress monitoring on physiological sensing pipelines that are already well established in stress computing.

This dominance is understandable. Physiological wearables align closely with common autonomic stress markers and are relatively easy to integrate into existing modeling pipelines. Studies such as Aktas et al. [12], Choi et al. [23], Jin et al. [14], Northrup et al. [19], and the dental monitoring work by Ludovichetti et al. [17] and Jaldin et al. [18] illustrate how wearable platforms continue to anchor the field. However, even within the review corpus, wearable dependence raises practical concerns around comfort, compliance, maintenance, and sensory burden, especially for younger children and neurodivergent users.

By contrast, clearly non-contact approaches remained limited. In the included studies, Chyan et al. [24] used speech-based stress detection, and Vural et al. [33] used facial-image analysis during physiotherapy game sessions. A small number of additional studies incorporated visual, audio, or contextual channels in hybrid configurations, including Loftness et al. [32], Zhang et al. [29], and Thierfelder et al. [31]. These studies show that non-contact and hybrid sensing are feasible in pediatric contexts, but they remain underused relative to wearable physiological monitoring.

Importantly, the evidence does *not* support the claim that the field contains a large or mature body of non-contact pediatric stress systems. Instead, the evidence suggests that non-contact methods are still emergent, often tested in constrained or task-specific settings, and rarely evaluated over extended real-world deployment. This is especially important because non-contact systems may offer practical advantages in comfort, compliance, and reduced sensor burden, particularly in classroom, therapy, or shared-environment contexts.

Another notable gap is the near absence of advanced contactless biosignal approaches such as remote photoplethysmography or other camera-based physiological estimation methods in pediatric field deployments. Although the review includes audio- and image-based studies, the broader contactless physiological sensing space remains largely unexplored in the pediatric corpus.

Future work should therefore move beyond wearable dominance by: (1) directly comparing wearable and non-contact approaches within the same pediatric populations and stress tasks; (2) testing audio- and vision-based systems in home, school, and therapy settings rather than only constrained tasks; and (3) exploring privacy-aware hybrid sensing pipelines that combine minimal physiological sensing with behavioral or contextual cues. Overall, non-contact sensing remains promising, but it is not yet a mature alternative to wearables in pediatric stress research.

### 4.2 Stress induction and validation protocols

The review confirms that pediatric stress studies use a wide range of induction and validation strategies, but without a shared methodological backbone. The included studies collectively span cognitive or academic challenges, social-evaluative tasks, game-based and interactive elicitation, naturalistic daily-life monitoring, and clinical or procedural stress contexts such as courtroom testimony and pediatric dentistry. This diversity is valuable because it reflects the real heterogeneity of child stress experiences. At the same time, it complicates comparison across studies.

Cognitive and school-related stressors were common, particularly in learning, homework, and performance-linked tasks. These were represented in studies such as Choi et al. [23], Jin et al. [14], Li et al. [30], Yao et al. [26], and Zhang et al. [29]. Social-evaluative paradigms also appeared in the included studies, including therapy-triggered sessions, robot-mediated interaction, mock interview structures, and courtroom-related stress monitoring [12, 27, 32, 35]. Game-based and interactive paradigms were particularly prominent in child-centered and autism-related work [22, 33, 37, 39, 43]. In parallel, several studies monitored stress in naturalistic settings without imposing a formal induction paradigm [15, 16, 41, 42].

What remains missing is standardization. Across the 34 included studies, the field still lacks a widely adopted pediatric equivalent of a canonical stress protocol. Stress labels were variously based on self-report, therapist or caregiver coding, event timing, physiological thresholds, or mixed schemes. This flexibility may be necessary in child research, but it also creates substantial heterogeneity in what counts as “stress,” how stress onset is defined, and how detection performance should be interpreted.

Validation methods were similarly heterogeneous. Physiological markers such as HRV, HR, EDA, ECG, and temperature remained central across many wearable studies. Self-report tools were widely used in older children and adolescents, but these are harder to rely on in younger children or in populations with communication differences. For that reason, observer- and caregiver-based labeling remained especially important in autism, therapy, and clinical contexts [12, 16, 41]. Mixed validation strategies were arguably the most defensible, but they were not consistently implemented.

This review therefore reinforces a central methodological point: pediatric stress detection does not yet have harmonized induction and validation standards. Future work should prioritize age-appropriate elicitation protocols, clearer annotation schemes, better reporting of label-generation logic, and stronger multimodal validation frameworks that do not depend on a single source of ground truth.

### 4.3 Contextual, demographic and cultural considerations

The 34 included studies provide an applied picture of the field, but important contextual and demographic limitations remain. The literature is no longer dominated purely by laboratory work; instead, it is distributed across school, therapy, clinic, mixed, home, courtroom, and procedural contexts. This is a strength, because it suggests that pediatric stress technology is increasingly being tested in environments that matter for real-world use. Even so, long-duration, ecologically rich, and cross-context studies remain comparatively limited.

School settings remained prominent in the review corpus, especially for academic stress, classroom-friendly sensing, and low-friction deployment [14, 22, 23, 32]. Clinical and therapy contexts were equally important, particularly in autism-related work, compulsive action monitoring, physiotherapy, and dental procedure monitoring [12, 17, 18, 33, 36]. Home-only studies existed, but remained relatively few, despite the fact that home life captures many of the everyday stressors that children experience [15, 16, 30, 42].

Age representation was more strongly concentrated among children under 13 years than among adolescents. This indicates that the review corpus is more strongly centered on child-focused and therapy- or school-linked stress technologies. Even so, children younger than about 8 years remain relatively underrepresented, and many systems still assume a level of self-report ability, task compliance, or device tolerance that may not generalize well to younger cohorts.

Neurodivergent populations, especially children with autism spectrum disorder, were strongly represented in the included studies. This is both a strength and a sign of where the field currently sees practical value. Studies such as Aktas et al. [12], Carlier et al. [37], the Coşkun studies [34, 43], Kumazaki et al. [27], Masino et al. [20], Northrup et al. [19], and Yu et al. [16] show sustained interest in autism-related stress contexts. However, broader neurodiversity beyond ASD remains much less represented, and reporting of ethnicity, socioeconomic background, and other demographic characteristics was often thin.

Cultural and contextual adaptation also remain limited. Some studies clearly reflected local context, for example through home-learning stress, school routines, or country-specific deployment settings but most systems were not deeply culturally tailored. This is a practical concern because stress expression, caregiver involvement, comfort with sensing, and acceptable intervention styles can vary across family structures, educational systems, and social environments. Future work should therefore take contextual fit more seriously, especially in multi-country, multilingual, or low-resource settings.

### 4.4 Methodological and technical rigor in stress detection

The included studies show a broad range of technical maturity, from early feasibility pilots and design-led intervention studies to more formal classification and repeated-measures analyses. Quantitative approaches remained dominant, but they were far from uniform. Some studies focused on predictive classification, others on physiological phase comparison, others on field feasibility or behavioral change, and others on mixed-methods intervention outcomes.

Conventional statistical and supervised learning approaches remained central across the review corpus. Several studies reported moderate to strong classification performance, often using HRV-, EDA-, or multimodal features [12, 14, 23, 29, 33]. At the same time, direct comparison across reported metrics remains difficult because performance was computed on different labels, tasks, sensing windows, and sample sizes. The strongest clearly charted performance values in the corpus were in the low 90% range.

Deep learning was present, but it was not dominant. It appeared explicitly in a small subset of studies, most notably Chyan et al. [24], Vural et al. [33], and Jin et al. [14]. These studies suggest that deep architectures can be useful for speech, image, or wearable-derived pattern recognition, but the review corpus does not support describing deep learning as a mature or standard approach in pediatric stress monitoring. Small sample sizes, narrow contexts, and limited openly reusable datasets remain major constraints.

Personalization was another promising but underdeveloped direction. Jin et al. [14] showed that subject-specific modeling can outperform more generic approaches. This is intuitively important in pediatric stress detection because children differ substantially in physiology, baseline arousal, developmental stage, and environmental stress exposure. However, personalized modeling also raises practical challenges around calibration burden, repeated data collection, and cross-context deployment.

Furthermore, a noticeable hardware and brand bias exists across the literature. A significant portion of the included wearable studies relied on a small set of commercial devices, notably the Empatica E4, Garmin wearables, and Shimmer3 GSR+ systems. While these platforms offer robust physiological sensing, this concentration limits the generalizability of standard feature-extraction thresholds to other open-source, custom, or lower-cost digital health architectures. It also complicates cross-study comparisons, as proprietary, device-specific signal processing and sampling algorithms often remain opaque. Over-reliance on a few high-end commercial platforms introduces vendor-dependence and poses cost and availability constraints that may hinder scalable, real-world deployment in broader pediatric care settings.

A deeper methodological issue is the inconsistency of evaluation design itself. The review still includes studies with very different validation logics, ranging from formal cross-validation to descriptive signal interpretation, therapist annotation, journaling, and feasibility-oriented field observation. This means that “performance” cannot be understood as a single comparable dimension across the corpus. For the field to progress, researchers will need stronger reporting consistency, clearer definition of labels and evaluation windows, and better separation between proof-of-concept feasibility and validated predictive performance.

### 4.5 Dataset challenges and opportunities

The extracted data reinforces a familiar problem in pediatric sensing research: datasets are central to progress, but the current ecosystem remains fragmented, small, and largely closed. Most studies in the review corpus relied on custom, study-specific datasets collected for a single task, setting, or target group. Only a very small number of entries were marked as open or partially public, and even those do not yet amount to a mature benchmark landscape.

This has several consequences. First, it limits reproducibility. When data remain private, cross-study comparison depends almost entirely on published summary metrics, which are often difficult to interpret across differing protocols. Second, it restricts model development. Many pediatric stress datasets are small and highly contextual, making it difficult to train robust, generalizable models, especially for multimodal or deep-learning pipelines. Third, it slows cumulative progress, because each new study effectively rebuilds its own data infrastructure.

The included studies also showed wide variation in annotation style. Some studies used self-report, others therapist or caregiver coding, others task-phase or event-based labels, and others mixed approaches. This flexibility may be necessary in pediatric populations, but it reduces interoperability across datasets. Even where physiological sensing was similar, stress labels could mean quite different things from one study to another.

There are still important opportunities here. The review corpus includes examples of dataset-minded work, particularly the Coşkun database study and a small number of partially public or shared datasets. These suggest that the field is beginning to recognize data infrastructure as a research contribution in its own right. Going forward, pediatric stress research would benefit from shared annotation conventions, stronger reporting of collection protocols, and ethical mechanisms for controlled reuse of sensitive child data. Progress in this area is likely to have an outsized impact on methodological quality across the field.

### 4.6 Intervention and mitigation strategies

Mitigation and intervention emerged as central themes in the literature. In the review corpus, a substantial subset of studies included some form of adaptive support, regulation aid, caregiver-facing feedback, or child-centered coping mechanism. This means the literature is not only about detecting stress; it also increasingly explores what to *do* with stress information once it is sensed.

Biofeedback remained one of the most common intervention patterns. Antle et al. [22] used interactive biofeedback in an anxiety-focused system; Li et al. [30] used biofeedback prompts in a homework context with parent involvement; Theofanopoulou et al. [25] used a smart toy for calming support; and Chaowadee et al. [28] used alert-based feedback in a learning setting. These studies point toward a broader shift from passive measurement to feedback-linked support.

Game-based and toy-based interventions also played a meaningful role, especially in child-centered and autism-related contexts [25, 37, 39]. These systems are especially relevant because they align intervention with engagement rather than treating coping as a purely clinical or instruction-based act. Caregiver- and parent-facing support systems were also more visible in the included studies [16, 20, 42]. This is important because many pediatric stress contexts are inherently relational, and effective intervention may depend as much on adult response as on child self-regulation.

Even so, the mitigation literature remains limited in duration and scale. Most intervention studies were short-term, context-bound, or feasibility-oriented. Strong longitudinal evidence remains scarce, and few studies assessed whether the benefits of stress-aware systems are sustained over time. Likewise, truly scalable multi-user systems that work across home, school, and therapy settings remain rare. The field has therefore made conceptual progress, but not yet broad translational progress.

### 4.7 Ethical, privacy, and scalability challenges in real-world implementation

Ethical and privacy concerns remain important but insufficiently operationalized in the pediatric stress corpus. Many studies mention assent, caregiver consent, or sensitivity of physiological data, but few deeply engage with explicit governance frameworks. This is a significant weakness because pediatric stress systems often process highly sensitive signals, including physiology, behavior, speech, and contextual life events. The problem is amplified in settings such as schools, clinics, therapy centers, and homes, where stress data can carry social and emotional consequences beyond mere technical classification. Continuous monitoring may affect how children are interpreted by adults, how parents respond, or how institutions act on sensed data. Yet most included studies focused on feasibility or efficacy rather than on downstream governance.

To transition from feasibility studies to responsible real-world deployment, future systems must implement concrete data governance and ethical safeguards. Based on the gaps identified in the review corpus, we recommend five explicit operational requirements: (1) *data minimization*, ensuring systems collect and retain only the specific signals required for the stated intervention; (2) *on-device or edge processing* to prevent the unnecessary transmission of raw physiological or behavioral data to cloud servers; (3) *age-appropriate and ongoing assent* mechanisms that provide child-understandable explanations of what is being sensed and why, parallel to formal caregiver consent; (4) clearly defined *retention and deletion policies* for pediatric data; and (5) strict *role-based access controls* governing who (e.g., clinician, teacher, parent, or child) is authorized to view specific resolutions of stress data.

Scalability is also a persistent challenge. Many promising systems depend on specialized sensors, therapist involvement, manual labeling, or controlled environments. These conditions may be appropriate for early-stage research, but they are harder to sustain in ordinary classrooms, public clinics, or low-resource homes. Although several studies involved applied deployments, very few addressed maintenance burden, affordability, calibration overhead, or long-term caregiver acceptance in a systematic way.

For the field to mature, privacy and scalability need to be treated as core design requirements rather than afterthoughts. Without these steps, high-performing prototypes may continue to stall before real-world adoption.

## 5 Implications for research and practice

### 5.1 Implications for researchers

**Standardize pediatric elicitation and validation.** The review still shows broad variation in stressors, labels, and validation strategies. Future work should develop age-appropriate protocols, multimodal validation logic, and clearer reporting standards.

**Prioritize ecological validity.** The review corpus is contextually diverse, but long-term, cross-context, in-the-wild studies remain limited. More home-school-clinic continuity is needed.

**Broaden developmental and demographic coverage.** The included studies are strongly child-focused and ASD-relevant, but younger children, broader neurodiversity, and more diverse demographic representation remain underdeveloped.

**Invest in pediatric data infrastructure.** Custom private datasets still dominate the field. Shared annotation conventions, better dataset documentation, and ethically reusable pediatric benchmarks are badly needed.

**Use pragmatic modeling strategies.** Lightweight and interpretable models may currently be more realistic for pediatric deployment than data-hungry deep models. Personalization is promising, but must be balanced against calibration burden.

**Expand non-contact and hybrid sensing.** Wearables still dominate. Audio-, vision-, and hybrid pipelines deserve more direct benchmarking in ecologically valid pediatric settings.

**Make mitigation a core design goal.** The review corpus shows that intervention is already an important part of the field. Future work should build more closed-loop, caregiver-aware, and longitudinally evaluated systems.

**Operationalize ethics and implementation.** Researchers should report not only accuracy or feasibility, but also privacy design, retention logic, data access rules, and deployment burden.

### 5.2 Implications for industry and practice

**Schools and child-facing services.** School-compatible tools should minimize burden, use understandable outputs, and support teachers or counselors without creating surveillance-heavy workflows.

**Healthcare and therapy settings.** Clinically aligned systems should emphasize interpretable signals, manageable alerting, and integration with existing care routines rather than stand-alone novelty.

**Devices and platforms.** Low-friction systems using minimal sensors, practical battery requirements, and privacy-preserving defaults are more likely to translate into real use.

**Caregiver-facing deployment.** Parent and caregiver support should be treated as a first-class pathway for intervention, especially in home-learning, autism, and daily-life stress settings.

**Implementation governance.** Real deployment requires not only an accurate model, but a concrete data governance framework. Developers and institutions must enforce data minimization, utilize on-device processing to protect raw biometrics, implement role-based access controls for teachers and caregivers, define strict data retention policies, and establish usable, child-appropriate communication for ongoing assent.

### 5.3 Research directions opened by this review

(1) **Pediatric protocol development.** Build reproducible, age-sensitive stress elicitation and validation frameworks for children.
(2) **Longitudinal cross-context cohorts.** Create multi-week and multi-setting pediatric stress datasets with shared annotation logic.
(3) **Non-contact and hybrid benchmarking.** Compare audio-, visual-, contextual-, and physiological approaches within the same pediatric populations.
(4) **Young-child methods.** Design lower-burden sensing, assent, and labeling tools for children below 8 years.
(5) **From detection to support.** Evaluate intervention systems over time using child, caregiver, and context-level outcomes rather than accuracy alone.
(6) **Ethics, privacy, and cost.** Treat governance, affordability, and sustainability as core scientific design dimensions.

## 6 Threats to validity

This scoping review has several limitations.

First, the review was restricted to English-language peer-reviewed literature. Relevant work in other languages or in grey literature may therefore have been missed. Furthermore, no formal review protocol was prospectively registered (e.g., on Open Science Framework). The absence of a registered protocol can increase the risk of selective reporting, and our exclusion of grey literature may omit unpublished negative results. We mitigated this risk by strictly defining the research questions, eligibility criteria, search strategy, and extraction categories prior to final screening and data charting, but we recommend prospective registration for future systematic updates in this domain.

Second, the field itself is highly heterogeneous. Included studies varied substantially in sensing modality, target age, deployment context, labeling strategy, and outcome reporting. This limited direct comparability and made some categorization decisions necessarily interpretive.

Third, several classification choices required judgment. In particular, boundaries between contact-based, non-contact, behavioral-only, app-based, and hybrid systems were not always explicit in the original studies. Similarly, the distinction between monitoring, mitigation, and broader supportive intervention was not always cleanly stated by authors. Although consistent coding rules were applied, some categories remain partly dependent on interpretation.

Fourth, reporting quality varied. Some studies provided detailed technical and participant information, while others offered only limited detail on sample composition, validation logic, or dataset accessibility. This affected the precision with which certain cross-study summaries could be made.

Fifth, some of the final conclusions depend on how categories are operationalized. For example, exact counts for non-contact vs. hybrid systems may shift depending on whether behavioral-only, audio-based, or multimodal contextual studies are grouped narrowly or broadly. For this reason, the review is strongest in identifying clear patterns and gaps rather than in asserting over-precise categorical boundaries where the source literature itself is inconsistent.

Despite these limitations, the review offers a structured and transparent synthesis of the 34 included studies and identifies key patterns, gaps, and priorities in the field.

## 7 Conclusion

Taken together, the 34 included studies mark meaningful progress across wearable sensing, game-based and caregiver-linked interventions, multimodal modeling, and emerging clinical applications such as courtroom and dental stress monitoring while also exposing the limits of the current evidence base. The Discussion (Section 4) interprets these patterns in detail; what the corpus ultimately shows is a field that has moved beyond pure detection but has not yet reached methodological or translational maturity.

Overall, pediatric stress monitoring is not only a sensing problem. It is also a developmental, contextual, ethical, and implementation challenge. Future progress will require sustained collaboration across engineering, psychology, education, healthcare, and design to create systems that are not only accurate, but also usable, responsible, and genuinely supportive of children’s well-being.

## Data availability statement

The original contributions presented in the study are included in the article/Supplementary Material, further inquiries can be directed to the corresponding author.
