# Supplementary material for: Digital technologies for non-invasive stress detection, monitoring, and mitigation in children and adolescents: a scoping review
Source: Front Digit Health. 2026 Jul 13;8:1867488. doi: 10.3389/fdgth.2026.1867488 (PMC13402478; doi:10.3389/fdgth.2026.1867488)
Supplement: Supplementary file 1 [file Supplementaryfile3.docx]

# Multimedia Appendix 3. PRISMA-ScR Checklist

The following checklist is adapted from the PRISMA-ScR (Preferred Reporting Items for Systematic reviews and Meta-Analyses extension for Scoping Reviews) guidelines (Tricco et al., 2018). It indicates where each item is addressed in the manuscript.

| Section | Checklist Item | Location in Manuscript |
| --- | --- | --- |
| TITLE | Identify the report as a scoping review. | Title page |
| ABSTRACT | Provide a structured summary including background, objectives, eligibility criteria, sources of evidence, charting methods, results, and conclusions. | Abstract |
| INTRODUCTION: Rationale | Describe the rationale for the review in the context of what is already known. | Introduction, Background |
| INTRODUCTION: Objectives | Provide an explicit statement of the review questions and objectives. | Introduction, Objectives |
| METHODS: Protocol and registration | Indicate whether a review protocol exists, and if and where it can be accessed. | Methods, Review Framework |
| METHODS: Eligibility criteria | Specify characteristics of sources of evidence (e.g., years considered, language, publication status). | Methods, Inclusion/Exclusion Criteria |
| METHODS: Information sources | Describe all information sources in the search (databases with dates of coverage). | Methods, Search Strategy |
| METHODS: Search | Present the full electronic search strategy for at least one database. | Multimedia Appendix 2 |
| METHODS: Selection of sources | State the process for selecting sources of evidence (screening, eligibility). | Methods, Study Selection |
| METHODS: Data charting process | Describe the methods of charting data from the included sources. | Methods, Data Extraction and Analysis |
| METHODS: Data items | List and define all variables for which data were sought. | Methods, Data Extraction and Analysis |
| METHODS: Critical appraisal of individual sources | If done, describe methods of critical appraisal. | Not applicable (scoping review) |
| METHODS: Synthesis of results | Describe the methods of handling and summarizing the data. | Methods, Data Analysis |
| RESULTS: Selection of sources | Provide numbers of sources screened, assessed for eligibility, and included, with reasons for exclusions. | Results, PRISMA Flowchart (Figure 1) |
| RESULTS: Characteristics of sources | Describe characteristics of included sources. | Results, Study Characteristics; Multimedia Appendix 1 |
| RESULTS: Results of individual sources | Present data from each study. | Multimedia Appendix 1 |
| RESULTS: Synthesis of results | Summarize main findings from the included studies. | Results, Thematic Synthesis |
| DISCUSSION: Summary of evidence | Summarize main results, linking to objectives. | Discussion, Section 4 |
| DISCUSSION: Limitations | Discuss limitations of the review process. | Threats to Validity |
| DISCUSSION: Conclusions | Provide a general interpretation of results and implications. | Conclusions |
| FUNDING | Describe sources of funding and support. | Funding/Conflicts of Interest |
