# Supplementary material for: Digital technologies for non-invasive stress detection, monitoring, and mitigation in children and adolescents: a scoping review
Source: Front Digit Health. 2026 Jul 13;8:1867488. doi: 10.3389/fdgth.2026.1867488 (PMC13402478; doi:10.3389/fdgth.2026.1867488)
Supplement: Supplementary file 3 [file Supplementaryfile2.docx]

# Multimedia Appendix 2. Database search strategies

The following database-specific queries were used to identify eligible studies. All searches were limited to publications in English between January 2013 and April 2026. The date of last search was April 2026.

## PubMed

("children"[MeSH Terms] OR child* OR adolescent* OR youth OR pediatric) AND (wearable OR "mobile applications" OR "non-invasive" OR "contactless" OR "ambient sensor*" OR "ubiquitous computing" OR "behavioral biometrics") AND (stress OR "stress detection" OR "stress monitoring" OR "stress regulation" OR anxiety OR emotion)

Filters: English, 2013/01/01 – 2026/04/01

## Scopus

TITLE-ABS-KEY (child* OR adolescent* OR youth OR pediatric) AND TITLE-ABS-KEY (wearable OR "mobile application*" OR non-invasive OR contactless OR "ambient sensor*" OR "ubiquitous computing" OR "behavioral biometric*") AND TITLE-ABS-KEY (stress OR "stress detection" OR "stress monitoring" OR "stress regulation" OR anxiety OR emotion)

AND PUBYEAR > 2012

## IEEE Xplore

("All Metadata":child* OR adolescent* OR youth OR pediatric) AND ("All Metadata":wearable OR "mobile application*" OR non-invasive OR contactless OR "ambient sensor*" OR "ubiquitous computing" OR "behavioral biometric*") AND ("All Metadata":stress OR "stress detection" OR "stress monitoring" OR "stress regulation" OR anxiety OR emotion)

Publication Year: 2013–2026

## ACM Digital Library

Abstract: (child* OR adolescent* OR youth OR pediatric) AND Abstract: (wearable OR "mobile application*" OR non-invasive OR contactless OR "ambient sensor*" OR "ubiquitous computing" OR "behavioral biometric*") AND Abstract: (stress OR "stress detection" OR "stress monitoring" OR "stress regulation" OR anxiety OR emotion)

Years: 2013–2026

## Google Scholar (supplementary screening)

children OR adolescents OR pediatric OR youth AND wearable OR mobile OR non-invasive OR contactless AND stress OR anxiety OR stress monitoring OR regulation

Date of search: April 2026
